# Improving training and support by improving our out of hours handover, Central and North West London NHS Foundation Trust

**DOI:** 10.1192/bjo.2021.507

**Published:** 2021-06-18

**Authors:** Mehmet Gez, Guang Xu

**Affiliations:** Central and North West London NHS Foundation Trust

## Abstract

**Aims:**

During out of hour handovers at St Charles Hospital – the two duty SHO (senior house officers) cover on site, whereas the on-call registrar and consultant are available to contact by phone. Some trainees may experience difficulties in contacting their seniors for support, or may not feel comfortable doing so. Trainees may also feel like they would benefit from being more informed of the hospital situation, or added learning and educational opportunities from the shift. The aim of this project was to improve the out of hours support for the on-call SHOs – which we hope to have positive short (such as improving confidence and performance) - and longer-term impacts (improving retention in the deanery and specialty).

**Method:**

The project proposed instating a 15-minute Zoom call at the start of each night shift (9:30pm) which involved the on-call team (SHOs, registrar, consultants and ideally bed managers). Firstly – a survey monkey questionnaire was sent to trainees to gain a baseline on how supported/informed/ease and learning opportunities for that shift. The project then piloted three separate Plan Do Study Act cycles of change and collected feedback from trainees after each cycle. Both qualitative feedback and quantitative feedback from trainees were collected in the Likert scale format after each PDSA cycle.

**Result:**

Results showed that a key benefit of this call is that any pressing issues can be brought up and addressed. Furthermore, for the benefit of the trainees, generally trainees felt more supported whilst they are on call, and got to know the fellow on call team. In addition, trainees reported feeling more at ease when calling their senior colleagues.

**Conclusion:**

It is particularly important for doctors to feel supported and informed during their on call shift, especially in the current climate, where there are fast changes and adaptations taking place due to the pandemic. By adding a short meeting at the beginning of each night shift, doctors in the hospital demonstrated an increase in feeling supported, informed and having educational opportunities during their on call shifts. In the long term, by addressing on call issues and making trainees feel more confident and supported during their shift, is likely to benefit and improve recruitment and retention.

